# Antiviral Defense and Innate Immune Memory in the Oyster

**DOI:** 10.3390/v10030133

**Published:** 2018-03-16

**Authors:** Timothy J. Green, Peter Speck

**Affiliations:** 1 Centre for Shellfish Research & Department of Fisheries and Aquaculture, Vancouver Island University, Nanaimo, BC V9R 5S5, Canada; 2 Department of Biological Sciences, Macquarie University, Sydney, NSW 2109, Australia; 3 College of Science and Engineering, Flinders University, GPO Box 2100, Adelaide, SA 5001, Australia; peter.speck@flinders.edu.au

**Keywords:** *Crassostrea*, OsHV-1, immune priming, interferon, RNAi, OsHV-1

## Abstract

The Pacific oyster, *Crassostrea gigas*, is becoming a valuable model for investigating antiviral defense in the Lophotrochozoa superphylum. In the past five years, improvements to laboratory-based experimental infection protocols using Ostreid herpesvirus I (OsHV-1) from naturally infected *C. gigas* combined with next-generation sequencing techniques has revealed that oysters have a complex antiviral response involving the activation of all major innate immune pathways. Experimental evidence indicates *C. gigas* utilizes an interferon-like response to limit OsHV-1 replication and spread. Oysters injected with a viral mimic (polyI:C) develop resistance to OsHV-1. Improved survival following polyI:C injection was found later in life (within-generational immune priming) and in the next generation (multi-generational immune priming). These studies indicate that the oyster’s antiviral defense system exhibits a form of innate immune-memory. An important priority is to identify the molecular mechanisms responsible for this phenomenon. This knowledge will motivate the development of practical and cost-effective treatments for improving oyster health in aquaculture.

## 1. Introduction

Molluscs are protostomes belonging to the Lophotrochozoa superphylum, the third major bilateral animal lineage after Deuterostomia and Ecdysozoa [1]. Information has long been scarce on the antiviral responses of molluscs. The reasons for this poor knowledge are multiple and mostly linked to the lack of continuous molluscan cell lines for virus propagation and characterisation [2]. Ostreid herpesvirus 1 (OsHV-1) belongs to the genus *Ostreavirus* from the family *Malacoherpesviridae* [3] and this virus has caused serious economic losses of the Pacific oyster, *Crassostrea gigas* [4,5,6,7,8,9]. Thus, OsHV-1 has prompted researchers to study the antiviral defense system of *C. gigas*. This data provides an evolutionary link for the development of antiviral defenses between Ecdysozoa and Deuterostomia. Several literature reviews describing cellular and humoral responses of marine molluscs against herpesvirus infection have recently been published [10,11,12], and these will be highlighted where appropriate. The focus of this review was to ask: how can our current understanding of the antiviral defenses of *C. gigas* (i) inform predictions on the evolutionary origins of the antiviral defense systems of animals, and (ii) reduce the economic impact of viral disease on shellfish aquaculture.

## 2. Antiviral Defense in the Animal Kingdom

Our understanding of innate antiviral immunity in animals is almost entirely defined by studies on vertebrates, insects, and nematodes [13,14,15]. One common theme between these animals is that the presence of double-stranded RNA (dsRNA) in virus-infected cells is a key inducer of the innate antiviral response [15,16,17]. The innate antiviral defense system of animals has pattern recognition receptors that sense dsRNA because most viruses, including herpesviruses that have a double-stranded DNA genome, produce significant amounts of dsRNA during their replication [18]. In general, animals have the cellular machinery to sense and process virus-derived dsRNA into short interfering RNAs (viRNAs) by a RNase III endonuclease [19]. When generated, viRNAs are loaded into a multi-subunit RNA-induced silencing complex (RISC), where they mediate sequence-specific cleavage of the viral RNA within the cell [19]. Insects and nematodes primarily utilize viRNAs to combat viral infection [20], whereas vertebrates cells instead use a protein-based defense called the type I interferon (IFN) system as the major innate antiviral response [15,21]. Viral infection of a vertebrate cell triggers the activation of a number of pattern recognition receptors (PRRs), with subsequent transcriptional activation of a family of IFN genes [15]. IFNs are secreted cytokines, released into the extracellular milieu where they bind specific receptors on the surface of infected and uninfected cells [15]. Receptor engagement activates signal transduction via the Jak/STAT pathway, leading to the transcription of hundreds of interferon-stimulated genes (ISGs) that work together to inhibit the cellular processes required by the virus to replicate and spread [22]. This narrow focus on vertebrates and model invertebrate species has given the impression that invertebrates use gene silencing by viRNAs to control virus replication, whereas vertebrates replaced this defense strategy with the IFN system [21,23].

The evolution of antiviral defense strategies in animals cannot be defined so simply. Firstly, insects also have a protein-based antiviral defense system that permits cell-to-cell communication of the immune response [24]. Virus-infected insect cells can secrete a peptide, Vago, to activate the Jak/STAT pathway and limit virus replication in neighbouring cells [25]. Insect cells detect replicating viruses using Dicer-2, which is central to the RNAi response, to activate Vago via a pathway dependent on TRAF and Rel2 [26,27]. This data suggests that, although structurally unrelated, Vago may have a function similar to vertebrate type I IFN cytokines [28]. Secondly, evidence is emerging that mature mammalian somatic cells can produce highly abundant viRNAs following infection with specific RNA viruses [29]. Many evolutionary questions arise from these studies on insects and mammals. Did the IFN and Vago pathways evolve from a common ancestor or arise via convergent evolution? How do the RNAi and IFN pathways cooperate, complement, or compensate for each other to successfully control viral infections in animals?

## 3. Antiviral Defense in the Oyster

*Crassostrea gigas* and OsHV-1 make an ideal model for studying the antiviral defenses in the Lophotrochozoa superphylum. Firstly, the genome of *C. gigas* was sequenced in 2012 [30], providing an opportunity for the discovery of evolutionarily conserved antiviral immune genes [10]. Secondly, reproducible laboratory-based experimental infection protocols have been developed using OsHV-1 from naturally infected oysters [31,32,33]. These tools have revealed *C. gigas* has a diverse set of antiviral defense pathways that are equipped with expanded and often novel receptors and adaptors [34,35].

**Figure 1:**
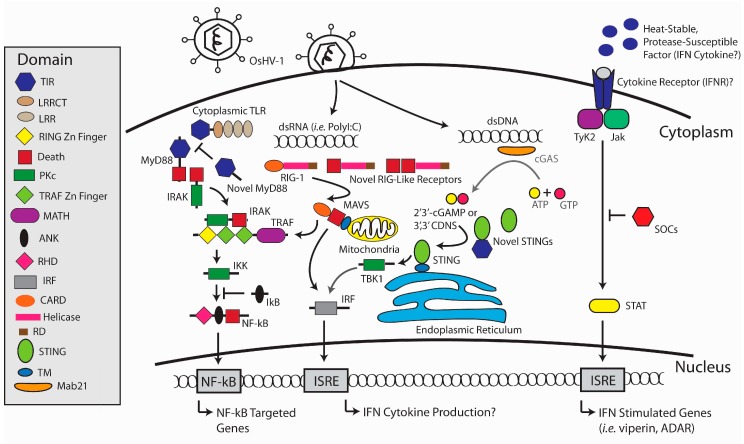
Conceptual diagram of the interferon-like antiviral response of *Crassostrea gigas* involving the TLR/NF-κB, RIG-1/MAVS, and putative cGAS/STING signaling pathways that result in the transcription of antiviral genes. The oyster genome encodes several novel toll-like receptors (TLRs) that lack transmembrane domains, implying they have a cytoplasmic function. These novel TLRs and downstream signaling adaptors are upregulated in response to OsHV-1 inoculation [34,36,52]. The oyster has a functional RIG-1 pathway that senses the presence of cytoplasmic dsRNA (i.e., polyI:C) and signals via downstream MAVS and TRAF adaptors [46]. The transcription factor IRF appears to function downstream of oyster MAVS and activates the IFN promoter and IFN stimulated response elements (ISRE) in mammalian cells [46,53]. Activation of IRF and NF-κB results in their translocation to the cell nucleus, leading to the transcription of antiviral genes. It is not currently known if oysters have a functional cGAS/STING-dependent antiviral response (pathway highlighted with grey arrows) [54]. The oyster genome encodes a STING homologue with three N-terminal transmembrane domains followed by a STING domain [10]. Oyster STING binds cyclic dinucleotides (CDNs) [55] and interacts with downstream TBK1 kinase [39]. Functional assays are required to determine if oyster cGAS binds cytosolic DNA and synthesizes CDNs. Note the unusual protein domains for RIG-like receptors and STING. Novel RIG-like receptors contain N-terminal death domains and novel STING either lack transmembrane domains or contain TIR domains [56]. TIR domain-containing proteins such as TLRs and interleukin-1 receptors are known to play key roles in innate immune signaling [57]. TLR, toll-like receptor; MyD88, myeloid differentiation primary response 88; IRAK, interleukin receptor-associated kinase; TRAF, TNF-receptor associated factor; IKK, IκB kinase; IκB, Inhibitor of κB; NF-κB, nuclear factor kappa-light-chain-enhancer of activated B cells; RIG-1, retinoic acid-inducible gene-1-like receptor; MAVS, mitochondria antiviral signaling protein; IFN, interferon; IRF, interferon regulatory factor; cGAS, cyclic GMP-AMP synthase; STING, stimulator of IFN genes; TBK1, tank binding kinase 1; STAT, signal transducer and activator of transcription; SOCS, suppressor of cytokine signaling; ISRE, IFN stimulated response element.

Transcriptome studies reveal an extensive set of *C. gigas* genes responding to OsHV-1 infection [34,35,36,37,38]. Highly activated genes include key components of the vertebrate type I interferon pathway (Figure 1). This includes homologs of cytoplasmic virus sensors, such as retinoic acid-inducible gene I (RIG-like receptors) and toll-like receptors (TLRs) that lack a trans-membrane domain [34]. Homologs of other interferon-signaling components include interferon-regulatory factors (IRFs), stimulator of interferon genes (STING), janus kinase (JAK), and signal transducer and activator of transcription (STAT) [34,37,39]. Several classic interferon stimulate genes (ISGs) are also upregulated in response to OsHV-1, including double-stranded RNA-specific adenosine deaminase (ADAR) and Viperin [36,37]. It was these data that led researchers to conclude that *C. gigas* might have an equivalent pathway to the vertebrate type I IFN pathway [34,37]. However, no obvious homologue of type I IFN cytokine (or arthropod Vago) has been identified in genomic datasets from *C. gigas* [10,34]. 

Efforts have been made to elucidate the molecular and cellular mechanisms involved in the oyster’s type I IFN-like pathway, and some recent progress has been made. Poly(I:C) is a synthetic double-stranded RNA (dsRNA) molecule that has been widely used as a viral mimic in mammalian and fish models to activate the type I interferon pathway [15,40,41]. Intramuscular injection of poly(I:C) and other long dsRNA molecules induce an antiviral response in *C. gigas* that hampers OsHV-1 replication [42,43,44,45]. Oysters have a RIG-1 like receptor (RLR) pathway that senses cytoplasmic poly(I:C) and transmits signals via mitochondrial antiviral protein (MAVS) to activate NF-κB [46]. *C. gigas* interferon regulatory factors (IRFs) appear to function downstream of MAVS and were able to activate the IFN-β and ISG response elements in mammalian cells [46]. Furthermore, the hemolymph from poly(I:C)-injected *C. gigas* contains a heat-stable, protease-susceptible factor that induces the transcription of ISGs in oyster hemocytes, such as Viperin [47]. Viperin is one of the few mammalian ISGs that have direct antiviral activity against a range of RNA and DNA viruses [48]. *C. gigas* Viperin inhibits dengue virus replication when over-expressed in a mammalian cell line [47]. 

Other antiviral defense pathways must also function in *C. gigas*, but have received less attention. Experimental infection of *C. gigas* with OsHV-1 has shown that surviving oysters can clear OsHV-1 DNA after 24 h post-inoculation [49]. Autophagy is thought to participate in OsHV-1 clearance and confers a protective role against the virus when induced by carbamazepine or starvation [50]. The OsHV-1 genome encodes many anti-apoptotic proteins that suppress apoptosis in *C. gigas* hemocytes and mantle tissue [43,51], despite components of the extrinsic apoptosis pathway being upregulated in response to OsHV-1 inoculation [34,36]. Other evolutionarily conserved antiviral pathways are yet to be investigated.

## 4. Evolutionary Origins of Antiviral Defense Systems

Studying the antiviral defenses of oysters has provided important insights into the evolution of innate immunity. It is currently debated whether vertebrates replaced the antiviral RNAi strategy with the type I IFN response [21,58]. Explanations for why vertebrates do not utilize RNAi are numerous. It has been suggested that the strong selective pressure imposed by viruses to inhibit RNAi may have accelerated the emergence of the vertebrate IFN response [21]. This argument is unlikely given *C. gigas* has an antiviral response with striking similarities to the vertebrate IFN response [34,37,42]. Together with genomic data from sponges (phylum: Porifera) [59,60,61], many key components of the IFN system appear to have ancient origins. Instead, absence of IFN-related genes in the genomes of insects (phylum: Arthropoda) and nematodes (phylum: Nematoda) appears to be a result of natural selection imposed by their viruses.

One of the important questions that remains to be investigated is whether *C. gigas* utilizes antiviral RNAi to control viral replication of DNA and RNA viruses. The *C. gigas* genome encodes the cellular machinery required for an antiviral RNAi defense system [10,62], but it is not known if this system is functional. Analysis of bivalve RNA-seq data has revealed numerous RNA and DNA viruses in *C. gigas* tissues [63]. Thus, identifying virus-derived siRNAs, by high-throughput sequencing, would confirm whether *C. gigas* utilizes this antiviral pathway to control these viruses. This knowledge could help address the question of why the type I IFN response supplanted siRNA antiviral pathway as the dominant antiviral innate response in vertebrates [15,23]. In mammalian cells, evidence is mounting to suggest that the type I IFN and RNAi antiviral systems are incompatible with each other [21]. The type I IFN response shuts down the RNA-induced silencing complex (RISC), and expression of dicer induces the type I IFN response [64,65]. It is possible that the antiviral RNAi system contributes little to the oyster’s defense. The oyster genome encodes dicer and ago2, but they are not upregulated in response to OsHV-1 infection [34].

The innate immune system relies on receptors to detect conserved determinants of microbial origin [66]. Activation of these receptors initiates signaling cascades that culminate in an effective immune response. The oyster’s immune system can distinguish between different types of infectious microorganisms and mount somewhat directed responses [36]. Several antiviral pathways in the oyster are equipped with expanded and often novel receptors and adaptors [35], which may assist in tailoring the oyster’s immune response to different types of pathogens. In particular, RIG-like receptors (RLRs), cyclic GMP-AMP synthase (cGAS), and stimulator of antiviral genes (STING) are all cytoplasmic viral receptors that belong to multi-gene families that have massively expanded due to tandem gene duplication and lineage-specific diversification [35,56]. These virus sensors, all known for their ability to activate the IFN response, often contain novel protein domains that may permit crosstalk with other immune pathways [35,56]. Although these virus receptors are classified as cytoplasmic, the novel domain architecture may enable different virus sensor isoforms to localize in different cellular compartments—such as endosome, cytosol, or nucleus—and thus provide spatial information regarding the viral infection [66]. Studies are warranted to determine whether the expansion of virus receptors assist *C. gigas* to discriminate different viral pathogens.

It also remains to be determined whether the cGAS-STING pathway exhibits an antiviral function in oysters, similar to its function in mammalian cells [67]. In mammals, evidence indicates that in most cell types the cytoplasmic DNA sensor cGAS is essential for induction of type I IFN response to DNA viruses [54,68]. cGAS binds dsDNA via its zinc ribbon domain and catalyzes the synthesis of a cyclic dinucleotide (CDN) called cGAMP [69]. cGAMP is a secondary messenger that binds directly to STING resulting in the conformational reorganization of the C-terminal tail (CTT) domain of STING that permits recruitment of the downstream TBK1 kinase [69]. TBK1 in turn phosphorylates the transcription factor IRF3, resulting in IRF3 translocation to the nucleus where it mediates transcription of IFN and other co-regulated genes [67,70]. The sources of cytoplasmic DNA that induce CDNs include the genomes of invading pathogens (i.e., intracellular bacteria and DNA viruses) and self-DNA that has leaked from the nucleus of host cells [67]. Bacteria are also capable of stimulating STING function by secreting STING activating CDNs (3′,3′ c-di-AMP, cAA) [71]. Phylogenetic analysis of cGAS and STING gene families showed that their origin could be traced back to the common metazoan ancestor and these two proteins have co-evolved in unison [55,69]. However, invertebrate cGAS homologues lack zinc ribbon domains probably required for DNA binding and invertebrate STING homologues lack the C-terminal tail (CTT) domain that permits recruitment of TBK1 [67,69]. Thus, it was proposed that invertebrate cGAS-STING pathway cannot sense cytosolic DNA to activate an antiviral response, but may be evolved in invertebrates as an antibacterial pathway [54,69,72]. Efforts to elucidate the molecular mechanisms of the oyster’s cGAS-STING pathway have revealed oyster STING homologs exhibit a robust ability to bind all naturally occurring CDNs, including cGAMP [55]. Furthermore, oyster STING interacts with TBK1 [39], despite lacking a recognizable CTT domain. Interesting, gene profiling experiments have identified oyster STING and TBK1 are induced by OsHV-1 inoculation and polyI:C stimulation [36,39,73], implying a potential antiviral role. Functional and biochemical assays are now required to determine whether oyster cGAS homologues sense cytosolic DNA to synthesize CDNs and if STING/TBK1 can activate oyster IRF resulting in the transcription of ISGs. It is highly likely that a far more complete understanding of the evolutionary origins of the cGAS-STING pathway will emerge from further characterization of this oyster pathway in response to immune stimulatory DNA (ISD) and poly(dA-dT) [74]. This knowledge would help address whether the cGAS-STING pathway first evolved as an antibacterial or antiviral response.

## 5. Innate Immune Memory and Antiviral Therapeutic Potential for Shellfish Aquaculture

The shellfish aquaculture industry is desperate to find ways to minimize the economic losses associated with viral diseases, such as OsHV-1 [75]. So far, oyster farmers have relied on traditional selective breeding to develop disease-resistant oysters [76] or changing farm husbandry techniques to reduce mortality by limiting contact time between the oyster and OsHV-1 [77,78,79,80]. These approaches all have inherent problems [81]. Developing disease resistant oysters takes years to achieve and is an expensive burden for industry [82], whereas changes to farm husbandry has the undesired economic consequence of reduced growth rates [76,79,80]. Observational experiments investigating the impact of OsHV-1 on aquaculture production have observed *C. gigas* that survived a mortality event in the past appeared to be resistant later in life [83,84]. It also appears this resistance can be passed from generation to generation. Female *C. gigas* exposed to non-lethal OsHV-1 infections transmit resistance to their offspring [85]. The molecular mechanisms that underpin this phenomenon are still to be discovered.

This phenomenon of improved survival upon secondary exposure to OsHV-1 is termed immune priming to distinguish the innate memory from the mechanistically different adaptive immunity in vertebrates [86,87]. Several studies have shown immune priming with ribonucleic acids of different structures (single or double-stranded) or various lengths (300 bp to 8000 bp) can elicit a protective antiviral response in *C. gigas* against subsequent challenge with OsHV-1 [42,43,44,45]. This protection is long-lasting, persisting for at least five months, and the enhanced survival was validated on a shellfish farm exposed to naturally reoccurring episodes of OsHV-1 [44]. Recent data also suggests that this protection could be maintained across generations. Offspring (D-veliger oyster larvae) produced from polyI:C-treated parents had double the chance of surviving exposure to OsHV-1 compared to control larvae [88]. PolyI:C has no effect on the survival of *C. gigas* [44,88]. Hence, the enhanced protection cannot be explained by natural selection. 

It is not currently known how the oyster’s immune system is able to store information regarding previously encountered virus antigens (i.e., dsRNA or polyI:C) to induce resistance upon secondary exposure to a virus. One intriguing possibility is an epigenetic mechanism, such as DNA methylation or non-coding RNA that elevates the constitutive gene expression of antiviral effectors [89,90]. This would allow the oyster’s innate immune system to have plasticity (i.e., memory), as evidenced by the significant upregulation of oyster ISGs for at least seven days following polyI:C stimulation [91]. Identifying the molecular mechanisms that underpin the oyster’s innate immune memory might enable a cost-effective therapeutic treatment to mitigate OsHV-1, which would have tremendous economic benefits for the shellfish aquaculture industry.

## 6. Conclusions

Research on antiviral defense of the oyster is still in its infancy. In the past five years, the development of laboratory-based experimental infection protocols combined with next-generation sequencing has enabled researchers to identify *C. gigas* has a complex antiviral response [34]. Oysters have a transcriptional response to viral infection that has striking similarities to the vertebrate type I IFN response [34,37]. Activating the oysters IFN-like response by injecting *C. gigas* with polyI:C results in the upregulation of ISGs that inhibit OsHV-1 replication [42]. As more studies begin to characterize the antiviral defense responses of the oyster, we see several important questions to be addressed. These include determining whether the oyster utilizes the siRNA pathway to prevent virus replication and does the expansion of cytoplasmic and novel virus-recognition receptors enable the oyster to tailor its antiviral response against specific viruses. The ability of oysters to recognize foreign DNA (ISD and un-methylated CpG motifs [74]) to induce an antiviral response has yet to be determined. Purifying and characterizing the cytokine (heat-stable, protease-susceptible factor [47]) that induces *C. gigas* ISG expression would also provide valuable new information on the evolutionary origins of the IFN pathway.

Research into the antiviral defense response of *C. gigas* is quickly moving from the basic characterization of evolutionarily conserved antiviral genes to the possibility of using therapeutic treatments to ‘immunize’ oysters against viral diseases. The concept of innate immune memory in *C. gigas* is supported by heterologous immune-priming experiments using polyI:C to elicit long-lasting protection against OsHV-1 [44]. PolyI:C and its derivatives are non-hazardous synthetic compounds currently produced as vaccine adjuvants [92]. Thus, multi-generation immune-priming using polyI:C holds great promise as a cost-effective strategy to breed entire crops of oysters with enhanced protection against OsHV-1 [88]. The process is safe and would be acceptable to consumers because oysters sold for human consumption would not have directly been exposed to polyI:C (parents injected with polyI:C). The credibility of polyI:C as a therapeutic would benefit greatly from description of the molecular mechanisms underpinning the oyster’s innate immune memory.
